# *Bacillus subtilis:* applications in the livestock and poultry industry in recent years: review

**DOI:** 10.5713/ab.25.0084

**Published:** 2025-05-19

**Authors:** Xin Zhang, Ji Cao, Shi Zhe Han, Zhi Lin Liu, Ze Lin Jia, Jia Yu Cui, Yu Hang Zhang, Hui Sheng Xiong, Xue Li Wang

**Affiliations:** 1 College of Animal Science and Technology, Inner Mongolia Minzu University, Tongliao, China; 2 Tongliao Horqin Left Back Banner Animal Disease Control and Prevention Center, Tongliao, China; 3 Tongliao Naiman Banner Animal Disease Control and Prevention Center, Tongliao, China

**Keywords:** *Bacillus subtilis*, Biological Characteristics, Livestock and Poultry Production, Mechanism of Action

## Abstract

Probiotics, as an alternative to antibiotics, are one of the better choices for ensuring product quality and food safety. *Bacillus subtilis* is often used in different forms, such as powder, granule, and liquid, in the livestock and poultry industry to produce feed and food additives and other products due to its advantages of high stability, low pathogenicity, high protein secretion capacity, and developed fermentation technology. This article reviews the biological characteristics, physiological functions, and effects on livestock and poultry production, as well as their mechanisms of action, to enhance the positive impact of the rational use of *Bacillus subtilis* as an additive in animal husbandry, such as feed and livestock food. In addition, this article also evaluates the potential of *Bacillus subtilis* to mitigate the negative effects of livestock and agricultural cultivation on the environment.

## INTRODUCTION

Since Europe banned the addition of antibiotics to animal feed, the search for alternatives to antibiotics has accelerated around the world [1]. Probiotics are safe, non-polluting, have no compound residues, and can only have a beneficial effect on the host when ingested in sufficient amounts [2]. However, when probiotics accumulate in the gut beyond a certain level, their effects become a double-edged sword. Probiotics have positive effects, such as improving intestinal health in livestock and poultry, enhancing gut barrier function, boosting immunity, and alleviating stress responses. But they also face challenges, including low survival rates, difficulties in colonization, individual tolerance variability, potential dysbiosis due to overuse, and the risk of pathogenicity [3]. Probiotics enhance host immunity and alleviate disease symptoms by balancing and enriching the host’s intestinal microbiota [4]. In recent years, the scope of application of *bacillus*-based probiotics in the livestock and poultry industry has gradually expanded and the scale has grown rapidly [5]. Safety-evaluated non-pathogenic *Bacillus cereus* strains demonstrate positive effects on livestock and poultry industry development [6]. The *Bacillus cereus* strain BC13 isolated from surrounding soils of broiler farms by Liu et al [7] exhibits probiotic potential. The BC13 strain demonstrates acid resistance, thermotolerance, and bile salt tolerance, with capabilities to degrade starch, cellulose, and proteins. These characteristics contribute to enhanced growth performance, antioxidant capacity, intestinal immunity, and gut microbiota composition in broilers. The *Bacillus cereus* strain GW-01, isolated from ovine rumen chyme, has demonstrated probiotic potential following safety evaluation [8]. These findings are consistent with the results reported by Liu et al. As the demand for livestock and poultry food continues to increase, it has become a challenge to ensure the quality of livestock and poultry food while improving animal productivity. *Bacillus subtilis* (*BS*), as a probiotic additive, can form endophytic spores, characterized by high-temperature resistance, acid and alkali resistance, strong resistance, etc. It can colonize the gastrointestinal environment such as low pH and bile salts, and can effectively improve the intestinal function of the animal, promote animal growth, and prevent diseases [9–11]. When used alone or in combination with other probiotics, *BS* exhibits superior performance in terms of gut microbiota and disease resistance due to its unique physiological characteristics and has broad development prospects [12,13]. A growing body of research suggests that [14], Some strains of *Bacillus* can promote the health of eukaryotic hosts, but whether they can become a health-promoting organism is far from enough to study the positive effects and beneficial characteristics related to specific microbial applications. It is also necessary to confirm the toxicity of specific microorganisms and whether their continued use will have negative effects on the host. When using *BS*, its safety testing should be strengthened, the dosage should be standardized, and unreasonable use should be avoided to prevent changes in bacterial invasion, which can seriously cause adverse effects such as disease, poisoning, or death to the host. Recent studies on the probiotic properties of *BS* have primarily focused on its specific domains, including plant growth [15,16], agricultural applications [17,18], bioprocessing [19], mildly saline-alkali soil microenvironments [20], livestock health [21,22], aquaculture [23], and pathogenic bacteria inhibition [24]. In contrast, this review provides a comprehensive analysis of *BS* applications in animal husbandry growth promotion and its multifaceted positive impacts on ecological systems, while emphasizing critical safety considerations for its use.

## CHARACTERIZATION AND FUNCTION OF BACILLUS SUBTILIS

### Biological characteristics

*BS* is a genus of *Bacillus*, an aerobic or facultative anaerobic Gram-positive rod bacterium, size (0. 8–1. 2) μm (1. 5–4. 0) μm, flagellum movement, no capsule, and can form spores [25,26]. *BS* is widespread in soil, water, plant, and animal body surfaces and decaying organisms, and can be easily isolated and cultured [27]. *BS* produces enzymes such as α-amylase, β-glucanase, protease, cellulase, and pectinase [28].

### Physiological functions

*BS* is one of the probiotics that are often added to feeds to improve the growth performance of livestock and poultry [29]. *BS* produces active substances such as mycobacteria, chlortetracycline, and short peptide, which are important in inhibiting pathogenic bacteria, significantly increasing the level of antibodies by activating leukocytes, and promoting the growth and development of host immune cells and organs to form acquired immunity [30,31]. *BS* increases tight junctions of IL-2, IL-4, IL-10, and IL-13 gene expression, promotes host-gut microbial interactions, improves intestinal flora, and affects the integrity of the intestinal barrier [32]. *BS* has the advantages of good palatability and pure natural, so it has a strong colonization ability in the animal intestine, regulates the balance of microbial colonies in the animal intestine, improves the level of animal intestinal health, and increases the immunity and antioxidant function of the body of livestock and poultry, and other biological functions [33,34]. Analysis using metabolomics techniques showed that supplementation of feed with *BS* increased the abundance of beneficial bacteria, probably due to the inhibition of the growth of pathogenic bacteria such as *Erysipelothrix* and *Shigella* spp. [35]. *Lactobacillus*, *Bifidobacterium*, *Bacillus*, and *Saccharomyces* are the main microbial species used as probiotics in animal feeding [36]. For example, dietary supplementation with *Bacillus licheniformis* increased villus height in the ileum: ratio of villus height to crypt depth [37]. Similarly, *BS* improves intestinal development and digestion by increasing the ratio of villus height to crypt depth in the jejunum, which in turn reduces intestinal disease [38]. The addition of a mixture of *Clostridium butyricum*, *BS*, and *Bacillus licheniformis* to diets increases body weight and average body weight of livestock and poultry [39]. As an efficient microbial ecology agent strain, *BS* can not only maintain high activity in livestock and poultry breeding production but also accelerate the conversion rate of feed, and improve the nutritional value of feed and the disease resistance of livestock and poultry [40].

## APPLICATION OF BACILLUS SUBTILIS IN LIVESTOCK RODUCTION

### *Bacillus subtilis* is closely related to livestock performance

The addition of probiotics in feed can prevent the colonization and reproduction of pathogenic bacteria in the intestinal tract of livestock and poultry, promote the conversion of feed and absorption of nutrients, and improve the growth performance of livestock and poultry [41]. *BS* is one of the intestinal microorganisms that can effectively improve intestinal function and maintain intestinal health, and adding it to feed rations has a positive effect on the growth performance of livestock and poultry [42].

Multiple studies confirm that supplementation with *BS* improves livestock growth performance, with optimal effects observed at specific dosages (e.g., 0.05%–0.1%). These effects are attributed to enhanced nutrient absorption and reduced diarrhea rates, highlighting the strain potential for improving feed efficiency in intensive farming systems [43]. Microbial ecology preparations from cellulase-producing *BS* (200 mg/kg) showed a better reduction of feed gain ratio and diarrhea rate in piglets and broilers compared to Colistin sulfate. The beneficial effects of *BS* as an alternative to antibiotics in improving livestock growth performance, in addition to the absence of adverse effects on the host, have been further demonstrated [44]. Choi et al [45] examined the intestinal contents of broilers after consumption of *BS*-supplemented diets using metabolomics techniques and quantitative analysis of *BS*. They noted that this diet promotes weight gain in chicks and helps boost intestinal anti-inflammatory capacity. Not only that, adding *BS* to the feed can also improve the production performance of Rex rabbits and improve the quality of their pelts [46]. It is widely recognized that probiotic combinations have a synergistic effect and are more effective than single-use [47]. Zou et al [48] found that the addition of a probiotic complex (0.05%) made from *BS*, *Clostridium butyricum*, and *Enterococcus faecalis* to the feed significantly increased the average daily gain and average daily feed intake of broilers. However, the addition of the complex (0.025%) improved the growth performance but not to a significant level. This may be related to the accumulation of probiotics in the gut, which can only have a positive effect on the organism if a certain amount of probiotics are accumulated in the gut. This study suggests that probiotics should be supplemented in adequate amounts to minimize farm losses.

The above studies have shown that the addition of *BS* to livestock and poultry feeds helps to improve the net protein utilization, nitrogen deposition, and nutrient digestibility of the ration, etc., and has a more desirable protein biological value [49,50]. Although adding *BS* to feed is beneficial to the growth of livestock and poultry, it is necessary to formulate a reasonable feeding management program according to the different stages of different animals when adding *BS*, to avoid the waste of additives caused by excessive additives as well as to bring certain pressure on the environment.

### *Bacillus subtilis* is relevant to livestock and poultry gut health

The animal intestinal tract is an important place for the digestion and absorption of nutrients, and it is also the largest immune organ of the animal organism [51]. *BS* has excellent biological properties and can secrete a variety of active substances, such as peptides and antimicrobial proteins, etc., which can better improve the intestinal flora and maintain the dynamic balance of microorganisms in the intestine [52].

This advantage is well-documented in the literature. For instance, Khan et al [53] inoculated the isolated *BS* strain was inoculated into sterile LB liquid medium at 1% (v/v) inoculum density. Cultivation was performed at 37°C with 200 rpm agitation for 15 h, followed by centrifugation at 2,400×g for 7 min to separate bacterial pellets from supernatant. For bacterial preparation, 1 kg starch was mixed with 360 mL bacterial suspension to achieve a final concentration of 3.6×10^10^ CFU/kg. The study found that supplementation of *BS* in the diet of broiler chickens significantly increased protease activity in the jejunum (p<0.05). Supplementation of *BS* (3.24×10^7^ CFU/kg) promoted the expression of the pro-inflammatory cytokine IL-1β in the kidney (p<0.01) and spleen (p<0.05). However, in the jejunum, supplementation with *BS* (1.08×10^7^ CFU/kg) increased the relative expression of Toll-like receptor (*TLR*) *2A* and *TLR 2B* (p<0.05). In contrast, the difference in gene expression of supplemented *BS* (3.24×10^7^ CFU/kg) was not significant (p>0.05). *TLRs* serve as fundamental components of the innate immune system, capable of recognizing diverse molecular patterns and consequently playing pivotal roles in host defense against pathogens [54]. To date, researchers have successfully identified over 20 *TLR* family members. These receptors exhibit specific recognition capabilities toward distinct pathogen-associated molecular patterns, enabling precise discrimination of diverse microbial invasions [55]. Approximately ten distinct *TLRs* have been identified in avian species (*Gallus gallus*), each demonstrating specific ligand recognition capabilities and functional involvement in *TLR* signaling pathways [56]. Among these, both *TLR1* and *TLR2* comprise A and B subtypes, with *TLR2* representing one of the most prevalent immune receptors in this family. The observed downregulation of TLR expression in this study may be due to a change in the timing of its regulatory effect by *BS* or due to the detection of Intestinal coccidiosis infections during the sampling process, which can also lead to a reduction in *TLR* over a short period. *BS* as a probiotic additive can regulate the intestinal microbiota, and an increase in the beneficial bacterial population in the intestinal tract can induce a decrease in the harmful bacterial population. To investigate the effect of supplementing the diet with *BS* on the immunity of piglets challenged with *Listeriae* infection, Garvey et al [57] showed that piglets supplemented with *BS* only had a 67% reduction in bacterial counts in the liver compared to piglets fed a regular diet (control), and a 49% reduction in splenic bacterial counts in piglets attacked with *Listeriae* infection and supplemented with *BS*. Visceral infection with Listeria infection affects intestinal morphology. *Listeriae* infection-only and *Listeriae* infection-attacked and supplemented with *BS* piglets showed in the ileal different increases and decreases ratio of villus height to crypt depth compared to the control group. This may be due to the ability of *BS* spores to germinate in the gut and secrete an anti-*Listeriae* infection that can be absorbed by the gut, thereby mitigating the spread of Listeria via the gut. This study unexpectedly revealed that visceral infection with *Listeriae* infection also affected intestinal morphology, and all intestinal morphologic parameters of *Listeriae* infection-infected piglets were similar to those of the control group, except for the height of the ileal villi. This suggests that *Listeria monocytogenes* infecting the ileum of piglets is more sensitive compared to the associated systemic inflammation and that it can also damage the intestinal epithelium more directly through invasion and colonization. *BS* can enhance nutrient absorption and feed utilization in livestock when added alone. When added to feed in combination with other microbial ecology agents, the efficacy can be better utilized. It has been reported that *BS* in combination with *Enterococcus faecium* better maintains the dynamic balance of the intestinal flora, resulting in a gradual increase in the number of beneficial microorganisms and a decrease in the number of harmful microorganisms. The effect of using the two together is greater than that of using them alone, which is more conducive to balancing the intestinal microbiota of laying hens [58]. This result is in agreement with the findings of Khan S. The combined use of probiotics has the potential to improve performance and maintain gut health in livestock and poultry, promoting the economic benefits of farming.

The addition of *BS* in feed can alleviate the stress response of livestock and poultry, maintain the intestinal health of livestock and poultry, enhance the immunity of livestock and poultry, and antioxidant and many other functions. Not only that, it can also increase the average daily weight gain of livestock and poultry, and reduce the ratio of the amount of feed consumed by livestock and poultry to gain weight of one kilogram [59,60].

### *Bacillus subtilis*’s assistance in improving the quality of meat and eggs

The addition of *BS* to feedstuffs has improved the quality of both meat and eggs of livestock and poultry. As a feed additive and one of the alternatives to antibiotics, the addition of Macleaya cordata extract is also helpful for meat and egg quality. Its main active ingredients are chelerythrine, sanguinarine, allocryptopine, and protopine, of which sanguinarine has anti-inflammatory effects [61,62].

It has been demonstrated in the literature, such as the study by Ren et al [63] which confirmed that the addition of a specific dose (0.2%) of *BS* (*BS* content ≥5.5×10^8^ CFU/g, purchased from Yantai Deep Sea Biotechnology) to the ration is more appropriate for the combined consideration of farm production and ration cost. The sub-prime egg rate in the *BS*-added group (0.45±0.1%) was lower than that in the group with the addition of macleaya cordata extract (0.84±0.07%), and the cholesterol content (2.23±0.24%) and high-density lipoprotein content (1.80±0.03%) of the *BS*-added group were lower than those of the macleaya cordata extract group with the addition of sterol (3.96±0.03%) and high-density lipoprotein content (2.27±0.03%) were lower. However, egg production rate, average egg weight, and feed intake were not significantly different from the addition of macleaya cordata extract. The experimental data showed that although the addition of macleaya cordata extract to the feed also favored meat and egg quality, the *BS*-added group had a lower sub-egg rate and better results. These effects are attributed to the fact that *BS* can secrete large amounts of peptides, oligosaccharides, phosphorus, and other active substances after entering the intestinal tract to maintain intestinal health and inhibit the colonization of pathogenic bacteria. The sheer force of muscle, a measure of muscle tenderness, is one of the indicators commonly used by consumers to evaluate pork quality when consuming pork. The PH final value affects the flavor of the meat, and meat with a lower final PH value is commonly referred to as “sour meat”. It has been shown that the addition of *BS* to the diet improves the metabolic processes in the longest back muscles of fattening pigs, and the final value of PH of pork in the *BS*-added group (5.93±0.07) was significantly lower (p<0.05) compared to the control group (5.76±0.03). The smaller the sheer force of the muscle, the more tender the muscle, the pork shear force in the *BS* group (20.64±1.30 N) was significantly lower (p<0.05) compared to the control group (25.41±1.39 N). However, there were no significant differences in water loss, yellowness values, and marbling of the muscles. This study confirms that *BS* improves meat quality and taste, which may be because the addition of *BS* to the feed improves the immune system of fattening pigs, allowing them to maintain their body health under conditions such as transportation (Group stress, heat stress, injury, restricted movement, and lack of rest can negatively impact animal welfare [64,65].), fasting (Pre-transport fasting in chicks adversely affects body weight gain and gastrointestinal tract development [66]. Similarly, pre-slaughter feed withdrawal in livestock leads to live weight loss and negatively impacts meat [64].), etc., and helps to keep the metabolic activity stable [67]. *BS* has been demonstrated to regulate intestinal microstructure and digestive enzymes; increase immunity against gut inflammation and infectious diseases; and enhance neurochemical activities via the bidirectional communication between the gut and brain, regulating stress responses under various rearing conditions [68]. As for egg quality, Souza et al [69] study on eggshells of 55–70 weeks laying hens showed that supplementation of *BS* in the diet increased the eggshell thickness of laying hens as compared to feeding the basal diet. This study confirms that egg breakage is associated with eggshell thickness and that increased calcium retention in laying hens is effective in reducing eggshell breakage [70]. Supplementation with the essential trace element Mn improves the expression of genes encoding proteoglycans and glycoproteins in the eggshell glands, which in turn increases the thickness of eggshells [71]. The study by Wang et al [72] demonstrated that dietary supplementation with specific doses of 30 mg/kg manganese and 5.0×10^9^ CFU/kg *BS* (Using *BS* powder containing 2×10^10^ CFU/kg, purchased from Puxing Biotechnology) to the feed improved the quality of goose eggs and the optimal combination of reproductive performance during the laying period in breeding geese. The combination of the two may have beneficial effects on gut health through favorable changes in the microbial community of the cecum. This contributes to the aspect of improved performance of breeding geese.

The above tests showed that *BS*, in addition to improving meat quality, had a better effect on egg quality and eggshell quality. Therefore, the addition of *BS* to feed rations plays a positive role in both meat quality and egg quality of livestock and poultry, improves the quality of livestock and poultry products, and promotes the high-quality development of the livestock and poultry industry.

### *Bacillus subtilis* is a facilitator of livestock immunity

*BS* can compete with pathogens, balance the intestinal flora, enhance the immunity of livestock and poultry, improve the growth performance of heat-stressed livestock and poultry, improve the recovery and regeneration process of damaged intestinal mucosa, and also increase the secretion of humoral immunoglobulin antibodies. Humoral immunoglobulins are mainly *IgA*, *IgG*, and *IgM* produced by B-cells, which are important parameters reflecting the state of humoral immunity in animals, and they play an important role in immune function and resistance to various infections [73–75].

Some studies have confirmed that supplementation of *BS* in the ratio can improve the growth performance of broilers by enhancing their immune function, regulating intestinal flora, and reducing the number of pathogens [76]. Sun [77] showed that for healthy white-feathered broilers of different ages, the addition of different doses of *BS* to the diet was able to increase serum immunoglobulin and immune organ indexes, enhance the immune function of broilers, reduce serum proinflammatory cytokines, inhibit the occurrence of inflammatory reactions, reduce the proportion of harmful microorganisms in the intestinal tract, improve intestinal morphology, and promote intestinal health. It has been reported that adding *BS* to feed can help increase the concentration of *IgA* in plasma. Lewton et al [78] have confirmed that specific changes in local and systemic immune markers and intestinal morphology in piglets were observed morphologically, suggesting that the addition of *BS* to feed is beneficial to the health of nursery pigs. *BS* can stimulate the systemic immune response and induce the production of *IgA*. The concentration of *IgA* in the overall plasma level increased by 20%, leading to an increase in the expression of antiinflammatory cytokine IL-10 in the jejunum. This research results showed an increase in plasma IgA concentration in the *BS*-added group compared to the control group (p<0.05). Dong et al [79] found that serum *IgA* and *IgM* were increased when *BS* alone or *lactobacillus* plantarum alone was supplemented in the diets of 70-day-old pigs, but no increase in *IgA* was found when *BS* was combined with *lactobacillus* plantarum. None of the three additions affected serum *IgG*. This may be because *IgM* is the first antibody to be produced at the beginning of the immune response, whereas *IgA* is the antibody that plays a key role in the mucosal immune system, which removes pathogenic bacteria utilizing innate nonspecific defense mechanisms. This suggests that *BS* stimulates a systemic immune response and plays an important role in inducing *IgA* production. This study highlights the potential of adding probiotics to feed to enhance mucosal immunity.

In conclusion, *BS* can improve the immunity of livestock and poultry, enhance the activity of immune cells, and effectively inhibit the number of harmful microorganisms in the intestinal tract and the occurrence of inflammation, which is beneficial to the health of livestock and poultry.

### *Bacillus subtilis*: boosting antioxidant capacity in livestock and poultry

Oxidative stress is caused by an imbalance between prooxidants and antioxidants, mediated by free radicals produced during physiological metabolic and pathological inflammatory processes, which can ultimately lead to cellular damage. Mitochondria can produce abundant reactive oxygen species, with superoxide being the most common one, Superoxide dismutase catalyzes the breakdown of superoxide into hydrogen peroxide and water and is a regulator of reactive oxygen species levels [80]. The production of free radicals in livestock and poultry is mainly carried out through two types of antioxidant systems: nonenzymatic antioxidant system and enzymatic antioxidant system, it can cause lipid peroxidation reactions and form lipid peroxides. The health level of livestock and poultry organisms is positively correlated with antioxidant capacity, the stronger the antioxidant capacity, the healthier the livestock and poultry organisms [81].

Cramer et al [82] research showed that feeding probiotics made from *BS* can reduce the oxidative stability of broiler chickens under chronic heat stress. Glutathione peroxidase, superoxide dismutase, and catalase levels can influence the stability of lipid oxidation in poultry meat during storage. Chronic heat stress reduced glutathione peroxidase (p< 0.0001) and superoxide dismutase (p<0.0001) activities in the breast muscle of broilers, which impaired the antioxidant defense system in broilers. Feeding probiotics made from *BS* significantly increased glutathione peroxidase (p = 0.0001) and it decreased superoxide dismutase (p<0.0001), suggesting an interaction between probiotic addition and heat stress (p = 0.001). After slaughter, the oxidation reaction in livestock and poultry meat has not yet stopped, in the main factor affecting the quality of meat is the lipid oxidation product malondialdehyde, which is formed by abundant unsaturated fatty acids through the reaction of free radicals. Malondialdehyde content in muscle is considered an important indicator of the degree of oxidation and safety of meat products during storage [83]. The addition of *BS* to the diet enhances the antioxidant function of the body by increasing the activity of certain antioxidant enzymes in the body. Zhu et al [84] showed that the addition of 200 mg/kg of *BS* to the diet increased the total antioxidant capacity, effectively increased the catalase content, and decreased the plasma malondialdehyde content in fattening pigs compared with the control group. This may be because *BS* produces free radical scavenging polysaccharides in the intestine of fattening pigs or induces the synthesis of Superoxide Dismutase and Catalase, which in turn accelerates the scavenging of oxides such as malondialdehyde [85]. It is also possible that *BS* exerts its antioxidant effects by enhancing the expression of antioxidant genes such as nuclear factor E2-related factor and Heme oxygenase-1, thereby enhancing the antioxidant capacity in the body [86]. This study provides theoretical support for the application of *BS* in swine production.

Therefore, *BS* can promote the production of antioxidant enzymes, improve the antioxidant capacity of livestock and poultry organisms, and reduce the incidence of related diseases caused by oxidative stress.

### Effects on blood biochemical indices of livestock and poultry

The protein content and other components in the blood can reflect the health status of the body, measurements of blood biochemical indices in livestock and poultry reflect changes in their physiological status and substance metabolism, such as stress and nutrient metabolism. The total protein content in serum is crucial for the state of livestock and poultry, reflecting the metabolic level of proteins in the body, and related to innate immunity in the body [87].

Plasma alanine aminotransferase activity is an important indicator of hepatic impairment [88]. In cases of impaired liver function, the activity of alanine aminotransferase in the blood is increased [89]. Wang et al [90] findings showed that the *BS* used in the experiment had no significant effect on the activity of alanine aminotransferase in plasma, indicating that *BS* is safe as a feed additive for laying hens. Blood is one of the most important indicators of the health status of livestock and poultry, and Oladokun et al [91] showed that supplementation of *BS* in feeds can have the same efficacy as the use of antibiotics in increasing plasma levels of sodium and chloride. This plays well into the role of *BS* as an alternative to antibiotic products. In addition to boosting the mineral content, *BS* can also effectively improve the serum biochemical indexes of livestock and poultry. Similar studies have shown that [92] the addition of *BS* to sheep and lamb diets significantly increased the concentrations of total protein, albumin, and globulin. Increased creatinine and glucose levels indicate activation of nitrogen, carbohydrate, and energy metabolism. In the serum of sheep and lambs, the decrease in total bilirubin and Cholesterol indicated a smooth lipid metabolism process and an increase in redox processes in the animals. This suggests that *BS* has the potential to maintain gut health in sheep and lambs. Under heat stress (average daily temperature-humidity index higher than 72), Choonkham et al [93] found that cows supplemented with *BS* in the diet had higher glucose levels than cows not supplemented with *BS*. This is attributed to the fact that *BS* can reduce indicators of negative energy balance by increasing glucose and decreasing beta-hydroxybutyric acid, and improve health indicators by keeping leukocytes and monocytes in the healthy range during the transition. Highlights the efficacy of *BS* in promoting increased digestion and absorption of nutrients.

The active substances contained in *BS* can improve the metabolism of organs such as the liver and kidney and the metabolism of proteins and other nutrients in livestock and poultry, which promotes the deposition of proteins and improves the synthesis and absorption of proteins in the diet of livestock and poultry. The addition of *BS* to livestock and poultry feed can better reduce the triglyceride and total cholesterol content of meat and eggs, which is conducive to meeting the increasing demand for meat and egg products with low saturated fat and low cholesterol [94,95].

### Contribute to the protection of the ecological environment

The current market demand for livestock and poultry-related products is constantly increasing, leading to the rapid development of the breeding industry. With the increase in breeding quantity and the expansion of breeding scale, the treatment of livestock manure and waste has become a major challenge. At present, a large amount of breeding waste has exceeded the carrying capacity of the environment, which has not only caused a certain degree of air pollution but also had a certain impact on the growth of crops.

Nitrogen fertilizer application releases large amounts of NH_3_ into the atmosphere, which is later deposited on land. synthetic nitrogenous fertilizer use rates are positively correlated with oxynitride and reduced nitrogen deposition on land [96]. Increased nitrogen deposition leads to global ecosystem biodiversity loss, surface water eutrophication, and soil acidification [97]. Sun et al [98] showed results of field trials that *BS* has good prospects in terms of mitigating the volatilization of NH_3_ from the land as a biofertilizer. The use of *BS* biofertilizer reduced NH_3_ volatilization by 44% compared to organic fertilizers. It also effectively reduced the abundance of biofertilizer *ureC* genes and increased the abundance of functional genes of bacteria *amoA* and *Comammox* and *ammonia-oxidizing bacteria* (*AOB*). The increase in its abundance can accelerate the transformation of organic nitrogen to inorganic nitrogen, thereby preventing the accumulation of ammonia. The application of *BS* as a biofertilizer demonstrates significant potential in mitigating NH_3_ volatilization from agricultural soils. This dual-benefit approach not only reduces environmental damage but also maintains high crop yields. In conclusion, *BS* demonstrates significant potential as a biofertilizer for mitigating ammonia (NH_3_) volatilization in agricultural systems. Liu et al [99] isolated and screened a *BS* strain from apple-continuous soil to study its effect on the continuous cropping of sweet tea in Pingyi. It was found that *BS* induced localized deformation, cell wall degradation, and concentration of cell contents in the pathogenic fungus hypha, and had antagonistic effects on *Fusarium moniliforme Sheld*, *F. oxysporum*, *Fusarium proliferatum*, and *Fungi Imperficti*. Compared with the control group, the use of *BS* as a fungal fertilizer could significantly promote the seedling growth of Pingyi sweet tea under continuous cropping conditions, increase soil enzyme activity, reduce the number of culturable fungi in the soil, and alleviate the soil continuous cropping barrier to a certain extent. This highlights the potential of *BS* as a bio-organic fertilizer in the research of crop continuous cropping obstacle prevention and control. As a commonly used plant inter-root growth promotion bacterium, *BS* can effectively optimize plant growth and yield. In agroecosystems, *BS* acts as an efficient nitrogen remover and maintains soil health through environmental remediation [100].

## DISCUSSION

*BS* is widely used in livestock and poultry production as a green and safe agent and is a better substitute for antibiotics. Simultaneously, this bacterium also has the functions of improving the growth performance of livestock and poultry, maintaining intestinal health, and promoting animal immunity, and has good development potential in clinical practice. In production and daily life, many bacteria are often used as probiotics, but due to the abuse, misuse, inadequate feeding management, and poor feeding environment of antibiotics, the invasiveness of bacteria has changed, leading to the phenomenon of causing animal diseases. In 2018, our team isolated one strain of *Bacillus cereus* from the spleen of sudden-death cattle, which was tested for virulence genetics and animal pathogenicity detection. The isolated strain was proved to be pathogenic *Bacillus cereus* [101]. Most *Bacillus cereus* has been used as a probiotic as an additive, but now it has been reported in many provinces and cities in China that *Bacillus cereus* is pathogenic or fatal to a variety of animals.

Therefore, to reduce the risk of harm to livestock and poultry, probiotics should be used in a scientific and standardized manner. When using *BS* as a feeding additive, the safety issues of this bacterium need to be further tested to enhance the safety testing of its adhesion to host tissues, pathogenic genes, and production of toxic metabolites. This review provides theoretical support for the feeding safety of livestock and poultry the high-quality development of livestock and poultry products and the protection of the ecological environment.

Therefore, *BS* has a positive impact on the ecological environment. For example, it reduces the pollution of livestock and poultry waste to the ecological environment, reduces the serious pollution and damage to the ecological environment caused by large-scale breeding, and promotes the healthy and sustainable development of animal husbandry.
